# Spinal muscular atrophy with respiratory distress type 1 (SMARD1): Report of a Spanish case with extended clinicopathological follow-up

**DOI:** 10.5414/NP300902

**Published:** 2015-12-28

**Authors:** Beatriz San Millan, Jose M. Fernandez, Carmen Navarro, Alfredo Reparaz, Susana Teijeira

**Affiliations:** 1 Department of Pathology,; 2 Department of Clinical Neurophysiology,; 3 Neonatal Intensive Care Unit; Complexo Hospitalario Universitario de Vigo (CHUVI), and; 4 Institute of Biomedical Research of Ourense·Pontevedra·Vigo (IBI), Vigo, Spain

**Keywords:** spinal muscular atrophy, SMARD-diaphragmatic palsy, respiratory distress, peripheral neuropathy

## Abstract

Background: Spinal muscular atrophy with respiratory distress type 1 (SMARD1) is a clinically and genetically distinct and uncommon variant of SMA that results from irreversible degeneration of α-motor neurons in the anterior horns of the spinal cord and in ganglion cells on the spinal root ganglia. Aims: To describe the clinical, electrophysiological, neuropathological, and genetic findings, at different stages from birth to death, of a Spanish child diagnosed with SMARD1. Patient and methods: We report the case of a 3-month-old girl with severe respiratory insufficiency and, later, intense hypotonia. Paraclinical tests included biochemistry, chest X-ray, and electrophysiological studies, among others. Muscle and nerve biopsies were performed at 5 and 10 months and studied under light and electron microscopy. Post-mortem examination and genetic investigations were performed. Results: Pre- and post-mortem histopathological findings demonstrated the disease progression over time. Muscle biopsy at 5 months of age was normal, however a marked neurogenic atrophy was present in post-mortem samples. Peripheral motor and sensory nerves were severely involved likely due to a primary axonal disorder. Automatic sequencing of *IGHMBP2* revealed a compound heterozygous mutation. Conclusions: The diagnosis of SMARD1 should be considered in children with early respiratory insufficiency or in cases of atypical SMA. Direct sequencing of the *IGHMBP2* gene should be performed.

## Introduction 

Spinal muscular atrophy with respiratory distress type 1 (SMARD1, OMIM #604320), also called diaphragmatic SMA, distal hereditary motor neuropathy type VI, or severe infantile axonal neuropathy with respiratory failure, is a clinically and genetically distinct form of spinal muscular atrophy type 1 (SMA1), first described by Mellins et al. in 1974 [1]. This is an extremely infrequent disease of autosomal recessive inheritance that results from irreversible degeneration of α-motor neurons in the anterior horns of the spinal cord and ganglion cells in the dorsal root ganglia (DRG). It leads to severe muscular atrophy of phrenic predominance causing early respiratory distress. Distal limb weakness subsequently occurs, with severe generalized hypotonia, areflexia, and progressive peripheral neuropathy. Prognosis is very poor and patients die of respiratory failure at an early age. The *IGHMBP2* gene, responsible for SMARD1, is located on chromosome 11q13, which encodes the immunoglobulin mu-binding protein 2 [2, 3, 4, 5, 6, 7, 8, 9]. 

Here we describe the clinical, electrophysiological and neuropathological findings, at different stages from birth to death, and the genetic studies of a Spanish child diagnosed with SMARD1 and a mutation in *IGHMBP2* [4]. Prenatal diagnosis was performed in a second pregnancy of the mother by chorionic villous biopsy at 11 weeks’ gestation. To our knowledge, this is one of the few cases with sequential examinations during life and post-mortem studies. 

SMARD1 should be considered as a differential diagnosis in every newborn with respiratory distress and diaphragmatic paralysis, even prior to muscle weakness onset. 

## Case report 

A 3-month-old girl was admitted to the hospital with respiratory difficulty, tachypnea and forced respiration with retractions, which had been noticed in an ordinary pediatric control. She was the first child of unrelated, young, and healthy parents without familial history of neuromuscular disorders or sudden infant death syndrome. The baby had been born by caesarean section at 34 weeks gestation due to intrauterine growth retardation (IUGR), decreased fetal movements, and abruptio placentae diagnoses. She weighed 1,550 g at birth (< 10^th^ percentile), with an Apgar score of 8/10, and presented neonatal respiratory distress immediately after birth, requiring mechanical ventilation and pulmonary surfactants for 3 days. 

On admission at 3 months, pulmonary auscultation revealed severe bilateral hypoventilation with crepitus. Chest X-ray suggested bronchiolitis and she received corticoids and oxygen without improvement. The disease progressed to severe respiratory insufficiency, requiring mechanical ventilation. Repeated efforts to wean her from ventilator resulted unsuccessful. 

Limb weakness with distal to proximal progression appeared shortly after the onset of respiratory insufficiency, with marked generalized hypotonia, areflexia, bilateral equinovarus foot deformities, and Achilles contractures. Tongue fasciculations were absent. 

Serologic and biochemical parameters, including creatinine kinase levels and cerebrospinal fluid analyses were normal. Results from other tests performed for infective and metabolic conditions were unrevealing. 

On electrophysiological examination, sensory potentials were abolished in the lower limbs and fingers. The only recordable potential was that of the median nerve (amplitude 2.6 µV, SCV 21 m/s) (Table 1). Concentric needle examination revealed severe partial denervation in all examined muscles of the upper and lower limbs. Complete denervation of the left hemidiaphragm was demonstrated. 

Electroencephalogram, brain magnetic resonance imaging (MRI), and cardiologic studies did not show abnormalities. Genetic analysis for Survival Motor Neuron (*SMN1*) gene was negative. Electron microscopy of skin and biochemical assays in cultured fibroblasts ruled out metachromatic and globoid cell leukodystrophies. 

**Figure 1.**
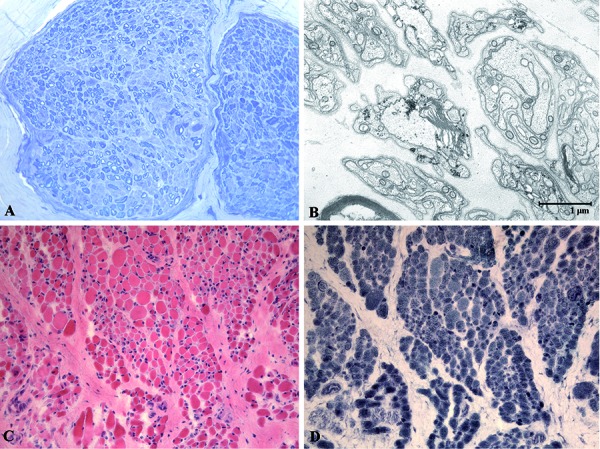
A: Semithin section of sural nerve biopsy at 5 months of age showing marked reduction of myelinated fiber density and endoneurial fibrosis, without onion bulbs, axonal sprouting, or inflammatory infiltrates. (Toluidine blue 200×). B: Demyelinated axons and Schwann cells arranged into bands of Büngner under electron microscopy examination of the sural nerve sample at 5 months. C: Muscle biopsy at 10 months of age (H & E 200×). Note marked peri and endomysial fibrosis and fatty infiltration with groups of small fibers and scattered hypertrophic fibers. D: NADH staining showing atrophic fibers with increased oxidative activity (NADH 200x).

Muscle and nerve biopsies were performed at 5 and 10 months of age. At 5 months muscle biopsy was nearly normal. However, sural nerve biopsy at that age demonstrated significant decrease in myelinated fiber density (Figure 1A) with no evidence of inflammation. At electron microscopy examination, no onion bulbs or axonal cluster formation were present. Microtubules and neurofilaments were normal. Giant axons, metachromasia, or lysosomal storage were absent (Figure 1B). 

**Figure 2.**
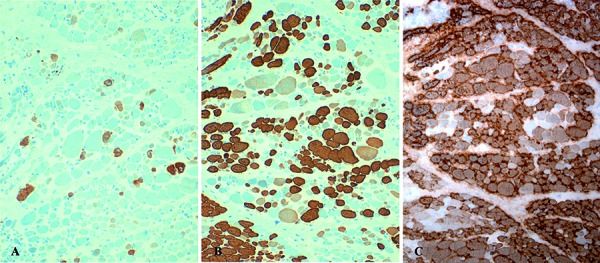
Immunohistochemical expression of developmental (dMHC), slow (sMHC), and fast (fMHC) myosin heavy chains in muscle biopsy at 10 months of age showing features of immature fetal phenotype. A: Developmental myosin predominates in atrophic and a few hypertrophic fibers (dMHC 200×). B: Slow HC myosin: hypertrophic fibers are characterized by their predominant expression; atrophic fibers also express slow HC. C: Fast MHC: atrophic fibers.

Electrophysiological examinations at 11 months of age demonstrated complete denervation in the most distal muscles (tibialis anterior, gastrocnemius, and foot muscles) and severely reduced interference pattern in the most proximal ones (vastus lateralis, deltoid, and facial muscles) (Table 1). At that age, marked atrophy of both type I and type II fibers was found in muscle biopsy (Figure 1C) with fibers showing prominent nuclei and increased oxidative activity, clearly demonstrative of a neurogenic process (Figure 1D) showing features of immature fetal phenotype (Figure 2). 

Despite therapeutic efforts, the condition of the child rapidly deteriorated, resulting in complete paralysis of limbs and trunk. In the end stage of the disease, tracheostomy and gastrostomy were required, and the baby developed recurrent pulmonary, urinary, and systemic infections. Autonomic manifestations subsequently occurred, including neurogenic bladder and cardiac arrhythmia. The child died of respiratory failure at 23 months of age. Permission to perform a necropsy was obtained. 

**Figure 3.**
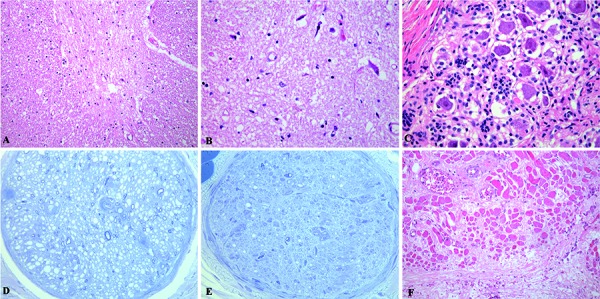
Post-mortem findings: A: Anterior horn of the spinal cord showing atrophy and significant reduction of motor neurons (H & E 200×). B: The remaining neurons presented chromatolysis and pyknosis (H & E 400×). C: Dorsal root ganglia with marked neuronal loss and abundant clusters of satellite cells’ nuclei (Nageotte nodules) (H & E 200×). D; E: Semithin sections. Remarkable nerve damage in sural and sciatic nerves, respectively, consisting of massive loss of myelinated fibers without signs of axonal regeneration or remyelination (Toluidine blue 100×). F: Severe fiber atrophy, fibrosis, and infiltration of diaphragmatic muscle sample (H & E 200×).

Post-mortem examination revealed bilateral pneumonic consolidation and pleural effusion. The spinal cord showed a reduced anterior spinal root diameter and prominent loss of motor neurons in the anterior horns (Figure 3A), extending from the cervical to the lumbar region. The remaining motor neurons displayed chromatolysis and pyknosis (Figure 3B). In DRG, significant neuronal loss was observed (Figure 3C), better depicted with S100 immunohistochemistry. The overall size of nerve cells was small with abundant clusters of satellite cells’ nuclei (Nageotte nodules). No abnormalities were present in the brain and midbrain motor nuclei. 

Severe axonal depletion was found in sural (Figure 3D) and sciatic (Figure 3E) nerve samples, with less than 50 – 60% of preserved fibers and prominent endoneurial fibrosis. Fibers showed thin myelin sheaths without signs of active demyelination or remyelination. Non-myelinated fibers were morphologically normal and basal lamina did not show abnormalities. Onion bulbs, axonal sprouts, or inflammatory infiltrates were absent. 

Muscle samples from the limbs, diaphragm, and intercostal muscles showed large groups of atrophic fibers with frequent nuclear clumps, massive adipose infiltration, and fibrosis, particularly in the diaphragm (Figure 3F). 

Further genetic studies were performed. Automatic sequencing of all 15 exons and flanking regions of *IGHMBP2* on 11q13-q21 revealed a compound heterozygous mutation R147X/C496X ([4], case no. 15). These mutations were demonstrated in the father and the mother respectively. 

Some years later, the mother became pregnant and underwent prenatal diagnostic tests in a chorionic villi biopsy at 11 weeks’ gestation, with her written informed consent. Prenatal molecular analysis in DNA extracted from chorionic villi samples proved the second fetus to be single heterozygote, harboring the paternal mutation. The pregnancy was continued, and a healthy girl was born at term and remains unaffected at 5 years of age. 

## Discussion 

SMARD1 is characterized by degeneration of anterior horn spinal motor neurons, leading to rapidly progressive neurogenic muscular atrophy of limbs and diaphragm [3]. Neurons on the DRG also degenerate, leading to an axonal neuronopathy, whereas involvement of the cranial nerves has only been reported in advanced cases [4]. Phenotype variability has been described even in siblings with identical mutations [10], and prenatal manifestations are not uncommon including IUGR, prematurity, and decreased fetal movements [11, 12]. An exhaustive clinicopathological review of 125 SMARD1 cases has been recently reported [13]. Our data, compared to those previously reported, are shown on Table 2. 

Some cases show early feeding difficulties or weak cry, but respiratory distress is the most common presenting feature. SMARD1 phenotype may be broader than initially considered [2, 10, 13], and few cases with milder phenotype and no evidence of diaphragmatic involvement until 4 years of age have been reported [2]. Respiratory distress resulting from irreversible phrenic denervation requires mechanical ventilation from early stages until death, which usually occurs in the first years of life. Unusual prolonged survival has been reported in some patients upon mechanical ventilation (from 4.5 to 21 years of age) due to exemplary care [14, 15]. 

Phrenic paralysis can be demonstrated on chest X-ray as diaphragmatic eventration and by electrophysiological studies, as in the present case. Plication of the diaphragm has been performed in several patients without success [16], and to date there is no effective drug therapy. At present, stem cell transplantation and gene therapy represent a potentially useful treatment strategy to ameliorate the SMARD1 phenotype [17, 18]. 

The child herein reported suffered her first respiratory distress episode early after birth, requiring mechanical ventilation. After the second episode, at 3 months of age, she remained ventilator-dependent until death at age 23 months. Diaphragmatic paralysis was demonstrated on the left hemidiaphragm by electrophysiological examination. Curiously, SMARD1 patients reported so far exhibited predominant right phrenic involvement, probably resulting from the pressure effect caused by the liver [13, 16]. 

Distal limb weakness, progressive generalized hypotonia, and foot deformities appeared after respiratory insufficiency. Quadriplegia, finger contractures, and fatty pads on the proximal phalanges resulting from adipose tissue infiltration, subsequently developed [4, 12, 19]. 

In addition to motor neuron involvement, neurons in the DRG are also involved in SMARD1, causing axonal sensory neuronopathy with distal to proximal progression. Significant neuronal loss and residual nodules of Nageotte at sites of destroyed neurons were clearly demonstrated in DRG at post-mortem examination. 

Corresponding to the clinical features, progressive loss of response for motor and sensory conduction velocities was observed, leading to an absent response after maximum stimulation in the end stage of the disease. 

Autonomic nervous system involvement is well documented in SMARD1, including excessive sweating, constipation, cardiac rhythm abnormalities, or sphincter control dysregulation [12, 20]. In the present case, cardiac arrhythmia and neurogenic bladder appeared late in the course of the disease. 

Pre- and post-mortem histopathological findings in nerves and muscles demonstrated the progression over time of SMARD1. Muscle biopsy at 5 months of age was considered normal, in contrast to significant loss of myelinated fibers observed in sural biopsy. A considerable degree of muscle involvement was noted at 10 months, with definite signs of neurogenic atrophy without signs of reinnervation. 

A marked progression of muscle damage was present in post-mortem samples, with the most severe damage in the diaphragm. 

Peripheral motor and sensory nerves were severely involved and the histopathological picture was most likely due to a primary axonal disorder, as there were no signs of demyelination/remyelination or onion bulb formation and the myelin in the remaining fibers was of normal thickness. There was no evidence of hypomyelination, thus excluding the diagnosis of congenital amyelinating or demyelinating neuropathy [21, 22, 23]. The spinal cord exhibited extensive loss of motor neurons in the anterior horns and DRG showed a marked reduction of ganglionic cells. 

Differential diagnosis of SMARD1 includes disorders displaying acute, early respiratory insufficiency and other causes of floppy infant syndrome. Some children were reported to have died from acute respiratory infection or sudden infant death syndrome [7, 24]. The main differential diagnosis should be established with SMARD2, a rare X-linked disorder with SMARD phenotype and mutations in *LAS1L* (ORPHA404521) [25]. Other possible diagnoses include SMA1 (Werdnig-Hoffmann disease). In contrast to SMARD1, proximal muscle weakness is the presenting sign of SMA1, and the respiration is compromised due to intercostal muscle involvement, as the disease progresses, causing paradoxical breathing and a bell-shaped thorax deformity. SMARD1 early involves phrenic muscle, without causing thoracic deformities. Peripheral sensory neuropathy, a hallmark of SMARD1, is not typically present in SMA1, although isolated cases have been reported [26]. Both SMA and SMARD1 children have normal intelligence and remain alert and attentive. 

Other causes of floppy infant syndrome must be considered, including glycogenosis type II (Pompe disease), or congenital myopathies (CM) with usually distinctive histopathological features. Congenital myotonic dystrophy type 1 presents with pronounced myopathic typical facies, arthrogryposis, and familial history [27]. Neonatal myasthenia gravis and congenital forms of myasthenic syndrome must be excluded, based on electromyographic findings and familial history. The lesional topography and fatal course of the disease in this patient were not suggestive of other disorders such as arthrogryposis multiplex congenita. 

IGHMBP2 is a ubiquitous ribosome-associated enzyme, that functions as a 5’-3’ RNA/DNA helicase [28]. The cellular pathomechanisms that result from *IGHMBP2* gene mutations are not well understood. Pathogenesis of motor neuron diseases as a group could be related with RNA metabolism [29]. *IGHMBP2* and *LAS1L*, both resulting in SMARD phenotypes, have roles in ribosomal biogenesis via processing of the 45S pre-rRNA [25]. However, a direct interaction between them has not been demonstrated [25]. 

In conclusion, we describe the neuropathological and electrophysiological findings of a Spanish patient with SMARD1, one of the more detailed cases with follow-up studies and post-mortem examination. The diagnosis of SMARD1, a more frequent disorder than suspected, should be considered in children with early respiratory insufficiency or in cases of atypical spinal muscular atrophy, even in the absence of overt diaphragmatic weakness [10]. SMARD1 diagnosis is of particular importance as it allows accurate genetic counselling and assists in the decision-making process for the initiation of mechanical ventilation of an affected infant [30]. Direct sequencing of the *IGHMBP2* gene should be performed if clinical and neuropathological features are compatible and *SMN1* mutation studies have been negative. Cases with normal *IGHMBP2* study should be screened for *LAS1L* mutations. Genetic counselling and prenatal diagnosis should be offered to all families with a familial history of SMARD1 in order to discuss the options and risks. 

## Acknowledgment 

We thank Tania Vazquez-Santos, Cristina Vazquez-Santos, and Soraya Barrera for technical assistance. This work was supported in part by Grants from the “Instituto de Salud Carlos III, ISCIII” (PI10/02628 and RD09/0076/00011); Xunta de Galicia (REGENPSI–2009/013) and by European Union funded projects Spain-RDR “Spanish Rare Disease Registries Research Network” (IRDiRC) and BIOCAPS (FP7/REGPOT-2012-2013.1, agreement nº 316265). 

## Conflict of interests 

The authors declare no conflict of interest. 

**Table 1.** Motor neurography performed in several nerves at 4, 11, and 16 months of age.

Nerve	Distal latency (ms)	Amplitude (µV)	MCV (m/s)	F-Lat
At 4 months of age				
Median	3.0	2.6	19.0	18.0
Right peroneal	5.2	0.3	14.0	Absent
Left tibial	5.0	1.2	13.0	45.7
Right phrenic	5.5	0.1	ND	ND
Left phrenic	5.4	0.1	ND	ND
At 11 months of age				
Median	3.4	0.6	16	18.8
Peroneal	4.3	0.2	13	ND
Tibialis	5.6	0.3	15	ND
Facial	4.3	0.3	ND	ND
Axilaris	2.7	0.5	ND	ND
At 16 months of age				
Median	4.9	0.1	17	ND
Facial	3.9	0.8	ND	ND
Axilaris	4.8	1.2	ND	ND

MCV = motor conduction velocity; F-Lat = F-wave latency; ND = not done.

**Table 2.** Clinical and histopathological findings.

	Porro et al., 2014 [13] (Review 1974 – 2014)	San Millán et al
Cases	127	Case report
Onset of respiratory symptoms	From birth – 3.5 years	First episode: at birth Second episode: 3 months
Age of death	1 hour – 5 years	23 months
Prenatal symptoms	None IUGR, Prematurity Low birth weight Oligohydramnios	IUGR Decreased fetal movements Abruptio placentae Low birth weight
Diaphragmatic involvement	Diaphragmatic paralysis: • Eventration in imaging • Right hemidiaphragm involvement • Respiratory weakness and recurrent pulmonary infections	Diaphragmatic paralysis: • Eventration on chest X-Ray and electromyopgraphy • Left hemidiaphragm involvement • Bronchiolitis • Death from pneumonia
Neuromuscular features	Hypotonia, areflexia, lingual fasciculations, weak cry, proximal and distal limb weakness, ankle and knee joint contractures, pes equinus, foot deformities, axonal sensory-motor neuropathy	Hypotonia, areflexia, weak cry, limb weakness with predominant distal involvement, pes equinovarus, Achilles and finger contractures, axonal sensory-motor neuropathy
CNS sensorial and autonomic	Cognitive dysfunction, language dysfunction, hypertension, urinary retention, feeding difficulties, dysphagia, slow peristalsis, arrhythmia, sweating, seizures, cardiovascular collapse	Neurogenic bladder Cardiac arrhythmia
Neuropathology	Spinal cord: • Degeneration and loss of anterior horn cells, atrophy of the nerve roots Skeletal muscle: • Neurogenic atrophy (limbs and diaphragm) Peripheral nerves: • Axonal degeneration and atrophy	Spinal cord: • Degeneration and loss of anterior horn cells, atrophy of nerve roots • Neuronal loss in dorsal root ganglia, Nageotte nodules Skeletal muscles (limbs, diaphragm, intercostal): • Severe neurogenic atrophy, particularly in the diaphragm Peripheral nerves: • Severe axonal depletion in sural and sciatic nerves, endoneurial fibrosis, thin myelin sheaths

## References

[1] Mellins RB Hays AP Gold AP Berdon WE Bowdler JD Respiratory distress as the initial manifestation of Werdnig-Hoffmann disease. Pediatrics. 1974; 53: 33–40. 4809192

[2] Grohmann K Wienker TF Saar K Rudnik-Schöneborn S Stoltenburg-Didinger G Rossi R Novelli G Nürnberg G Pfeufer A Wirth B Reis A Zerres K Hübner C Diaphragmatic spinal muscular atrophy with respiratory distress is heterogeneous, and one form Is linked to chromosome 11q13-q21. Am J Hum Genet. 1999; 65: 1459–1462. 10521314 10.1086/302636 PMC1288300

[3] Grohmann K Schuelke M Diers A Hoffmann K Lucke B Adams C Bertini E Leonhardt-Horti H Muntoni F Ouvrier R Pfeufer A Rossi R Van Maldergem L Wilmshurst JM Wienker TF Sendtner M Rudnik-Schöneborn S Zerres K Hübner C Mutations in the gene encoding immunoglobulin mu-binding protein 2 cause spinal muscular atrophy with respiratory distress type 1. Nat Genet. 2001; 29: 75–77. 11528396 10.1038/ng703

[4] Grohmann K Varon R Stolz P Schuelke M Janetzki C Bertini E Bushby K Muntoni F Ouvrier R Van Maldergem L Goemans NM Lochmüller H Eichholz S Adams C Bosch F Grattan-Smith P Navarro C Neitzel H Polster T Topaloğlu H Infantile spinal muscular atrophy with respiratory distress type 1 (SMARD1). Ann Neurol. 2003; 54: 719–724. 14681881 10.1002/ana.10755

[5] Guenther UP Schuelke M Bertini E D’Amico A Goemans N Grohmann K Hübner C Varon R Genomic rearrangements at the IGHMBP2 gene locus in two patients with SMARD1. Hum Genet. 2004; 115: 319–326. 15290238 10.1007/s00439-004-1156-0

[6] Guenther UP Varon R Schlicke M Dutrannoy V Volk A Hübner C von Au K Schuelke M Clinical and mutational profile in spinal muscular atrophy with respiratory distress (SMARD): defining novel phenotypes through hierarchical cluster analysis. Hum Mutat. 2007; 28: 808–815. 17431882 10.1002/humu.20525

[7] Bertini E Gadisseux JL Palmieri G Ricci E Di Capua M Ferriere G Lyon G Distal infantile spinal muscular atrophy associated with paralysis of the diaphragm: a variant of infantile spinal muscular atrophy. Am J Med Genet. 1989; 33: 328–335. 2801766 10.1002/ajmg.1320330309

[8] Novelli G Capon F Tamisari L Grandi E Angelini C Guerrini P Dallapiccola B Neonatal spinal muscular atrophy with diaphragmatic paralysis is unlinked to 5q11.2-q13. J Med Genet. 1995; 32: 216–219. 7783173 10.1136/jmg.32.3.216 PMC1050321

[9] Basel-Vanagaite L Taub E Drasinover V Magal N Brudner A Zlotogora J Shohat M Genetic carrier screening for spinal muscular atrophy and spinal muscular atrophy with respiratory distress 1 in an isolated population in Israel. Genet Test. 2008; 12: 53–56. 18298318 10.1089/gte.2007.0030

[10] Joseph S Robb SA Mohammed S Lillis S Simonds A Manzur AY Walter S Wraige E Interfamilial phenotypic heterogeneity in SMARD1. Neuromuscul Disord. 2009; 19: 193–195. 19157874 10.1016/j.nmd.2008.11.013

[11] Kaindl AM Guenther UP Rudnik-Schöneborn S Varon R Zerres K Schuelke M Hübner C von Au K Spinal muscular atrophy with respiratory distress type 1 (SMARD1). J Child Neurol. 2008; 23: 199–204. 18263757 10.1177/0883073807310989

[12] Eckart M Guenther UP Idkowiak J Varon R Grolle B Boffi P Van Maldergem L Hübner C Schuelke M von Au K The natural course of infantile spinal muscular atrophy with respiratory distress type 1 (SMARD1). Pediatrics. 2012; 129: e148–e156. 22157136 10.1542/peds.2011-0544

[13] Porro F Rinchetti P Magri F Riboldi G Nizzardo M Simone C Zanetta C Faravelli I Corti S The wide spectrum of clinical phenotypes of spinal muscular atrophy with respiratory distress type 1: a systematic review. J Neurol Sci. 2014; 346: 35–42. 25248952 10.1016/j.jns.2014.09.010

[14] Pierson TM Tart G Adams D Toro C Golas G Tifft C Gahl W Infantile-onset spinal muscular atrophy with respiratory distress-1 diagnosed in a 20-year-old man. Neuromuscul Disord. 2011; 21: 353–355. 21353777 10.1016/j.nmd.2011.02.005 PMC3085694

[15] Hamilton MJ Longman C O’Hara A Kirkpatrick M McWilliam R Growing up with spinal muscular atrophy with respiratory distress (SMARD1). Neuromuscul Disord. 2015; 25: 169–171. 25454169 10.1016/j.nmd.2014.10.005

[16] Kaindl AM Guenther UP Rudnik-Schöneborn S Varon R Zerres K Gressens P Schuelke M Hubner C von Au K [Distal spinal-muscular atrophy 1 (DSMA1 or SMARD1)]. Arch Pediatr. 2008; 15: 1568–1572. 18804971 10.1016/j.arcped.2008.07.014

[17] van der Pol WL Talim B Pitt M von Au K 190 th ENMC international workshop: Spinal muscular atrophy with respiratory distress/distal spinal muscular atrophy type 1: 11-13 May 2012, Naarden, The Netherlands. Neuromuscul Disord. 2013; 23: 602–609. 23726377 10.1016/j.nmd.2013.04.004

[18] Simone C Nizzardo M Rizzo F Ruggieri M Riboldi G Salani S Bucchia M Bresolin N Comi GP Corti S iPSC-Derived neural stem cells act via kinase inhibition to exert neuroprotective effects in spinal muscular atrophy with respiratory distress type 1. Stem Cell Rep. 2014; 3: 297–311. 10.1016/j.stemcr.2014.06.004 PMC4176534 25254343

[19] Rudnik-Schöneborn S Stolz P Varon R Grohmann K Schächtele M Ketelsen UP Stavrou D Kurz H Hübner C Zerres K Long-term observations of patients with infantile spinal muscular atrophy with respiratory distress type 1 (SMARD1). Neuropediatrics. 2004; 35: 174–182. 15248100 10.1055/s-2004-820994

[20] Mohan U Misra VP Britto J Muntoni F King RH Thomas PK Inherited early onset severe axonal polyneuropathy with respiratory failure and autonomic involvement. Neuromuscul Disord. 2001; 11: 395–399. 11369191 10.1016/s0960-8966(00)00210-8

[21] Simonati A Fabrizi GM Pasquinelli A Taioli F Cavallaro T Morbin M Marcon G Papini M Rizzuto N Congenital hypomyelination neuropathy with Ser72Leu substitution in PMP22. Neuromuscul Disord. 1999; 9: 257–261. 10399754 10.1016/s0960-8966(99)00008-5

[22] Warner LE Mancias P Butler IJ McDonald CM Keppen L Koob KG Lupski JR Mutations in the early growth response 2 (EGR2) gene are associated with hereditary myelinopathies. Nat Genet. 1998; 18: 382–384. 9537424 10.1038/ng0498-382

[23] Mandich P Mancardi GL Varese A Soriani S Di Maria E Bellone E Bado M Gross L Windebank AJ Ajmar F Schenone A Congenital hypomyelination due to myelin protein zero Q215X mutation. Ann Neurol. 1999; 45: 676–678. 10319895 10.1002/1531-8249(199905)45:5<676::aid-ana21>3.0.co;2-k

[24] Bove KE Iannaccone ST Atypical infantile spinomuscular atrophy presenting as acute diaphragmatic paralysis. Pediatr Pathol. 1988; 8: 95–107. 3399458 10.3109/15513818809022282

[25] Butterfield RJ Stevenson TJ Xing L Newcomb TM Nelson B Zeng W Li X Lu HM Lu H Farwell Gonzalez KD Wei JP Chao EC Prior TW Snyder PJ Bonkowsky JL Swoboda KJ Congenital lethal motor neuron disease with a novel defect in ribosome biogenesis. Neurology. 2014; 82: 1322–1330. 24647030 10.1212/WNL.0000000000000305 PMC4001186

[26] Rudnik-Schöneborn S Goebel HH Schlote W Molaian S Omran H Ketelsen U Korinthenberg R Wenzel D Lauffer H Kreiss-Nachtsheim M Wirth B Zerres K Classical infantile spinal muscular atrophy with SMN deficiency causes sensory neuronopathy. Neurology. 2003; 60: 983–987. 12654964 10.1212/01.wnl.0000052788.39340.45

[27] Harper PS Monckton DG Myotonic dystrophy In: Engel AG, Franzini-Amstrong (eds). Myology. New York: McGraw-Hill 2004 p. 1039-1076.

[28] Guenther UP Handoko L Laggerbauer B Jablonka S Chari A Alzheimer M Ohmer J Plöttner O Gehring N Sickmann A von Au K Schuelke M Fischer U IGHMBP2 is a ribosome-associated helicase inactive in the neuromuscular disorder distal SMA type 1 (DSMA1). Hum Mol Genet. 2009; 18: 1288–1300. 19158098 10.1093/hmg/ddp028

[29] Bertini E Houlden H Defects of RNA metabolism in the pathogenesis of spinal muscular atrophy. Neurology. 2014; 82: 1298–1299. 24647031 10.1212/WNL.0000000000000321

[30] Sangiuolo F Filareto A Giardina E Nardone AM Pilu G Pietropolli A Bertini E Novelli G Prenatal diagnosis of spinal muscular atrophy with respiratory distress (SMARD1) in a twin pregnancy. Prenat Diagn. 2004; 24: 839–841. 15503272 10.1002/pd.964

